# Review of "Witches, Westerners, and HIV: AIDS and Cultures of Blame in Africa" by Alexander Rodlach

**DOI:** 10.1186/1742-6405-4-5

**Published:** 2007-03-07

**Authors:** Kearsley A Stewart

**Affiliations:** 1 Department of Anthropology, Northwestern University, Evanston, IL 60208 USA

This easy-to-read, scrupulously researched, and fascinating book addresses two critical, but stubborn problems which threaten to reduce the effectiveness of many externally-funded HIV/AIDS prevention and treatment programs in Africa. First is the reluctance by biomedical and public health practitioners to recognize the essential value of qualitative and ethnographic data for the success of AIDS intervention programs in Africa. Second is the challenge of explaining the culturally coherent logic behind the seemingly irrational and contradictory views of Africans who blame sorcery and witchcraft for the HIV/AIDS epidemic. While this book will not completely solve both of these entrenched problems, it is a powerful statement about the value of systematically studying local explanatory models of the AIDS epidemic and offers a convincing and fine-grained analysis of the African quest to explain and account for personal misfortune in a time of significant social and economic uncertainty.

Rodlach's description of AIDS-related sorcery accusations and conspiracy theories is rooted in over a decade of work and research in rural and urban Zimbabwe. Although the book focuses specifically on Zimbabwe, there are many similarities to accounts of the AIDS epidemic elsewhere throughout southern and central Africa. His methodology draws on the usual skill-set of a qualitative researcher (key-informant interviews, observation, focus-groups, printed media and archival research, etc) and his theoretical framework rarely veers far from the standard anthropological literature on witchcraft in Africa (Douglas, Herdt and Stoller, Comaroff and Comaroff, Geschiere, Ashforth); but his extended residence in Zimbabwe as a practicing priest in the local Catholic Church clearly distinguishes him from other social scientific researchers. Fluent in Ndebele, Rodlach gained the confidence of his informants as he ministered to their suffering; this trust made possible his frank discussions about deeply-held and often hidden explanations of AIDS misfortune.

The chapters on conspiracy theories will be of most interest to clinicians, medical researchers, and other healthcare providers as these professionals feature prominently in local explanations of the origin of HIV and the spread of AIDS. In fact, these are Rodlach's strongest chapters because they are based on a broad and diverse range of evidence and, unlike accusations of sorcery and witchcraft, conspiracy theories are publicly discussed thus revealing a shared consensus and logic. The origin of HIV is sometimes attributed to "clever" (meaning selfish) western researchers and their Zimbabwean colleagues whose experiments on HIV in primates went awry and infected the human population. Rodlach suggests that this local disgust of biomedical research originates in strong taboos against transgressions of primate-human boundaries, the researchers' failure to adhere to other local knowledge and traditions, and the suspect intentions of anyone who is personally enriched by biomedical research. Clearly, this is fertile ground for the emergence of a conspiracy theory that holds biomedical research responsible for the spread of HIV. Rodlach then situates these seemingly irrational beliefs in the context of colonial medical practices during the Spanish Influenza epidemic of 1918–1919, rampant iatrogenic morbidity and mortality, and the poisoning of maize flour by white Rhodesian farmers. This serves to demonstrate that that which is seemingly irrational is, in fact, a logical interpretation of the origin of HIV/AIDS against these historical circumstances.

Also of interest to healthcare practitioners is an explanation of why even literate and educated Zimbabweans can simultaneously hold both biomedical and conspiracy explanations for the origin and transmission of HIV. Elite and healthy civil sector professionals who initially discredit sorcery charges may later invoke these same explanations to account for why they are diagnosed with AIDS when others are not. To explain this, Rodlach argues that causality is ambiguous. Because a variety of biomedical and social factors can lead to the symptoms of AIDS, and these causal factors can be variously categorized as remote, intermediate, proximate, or ultimate, it is therefore possible for people to hold multiple and even contradictory explanations of HIV. Clearly, the most effective AIDS education programs engage with, rather than deny, these multiple explanations. However, the link between beliefs and behaviors was not substantially addressed in the book, and the cursory discussion of the A-B-C controversy needed more critical attention. Despite these small shortcomings, Witches, Westerners, and HIV is an engaging discussion of a difficult and complex topic, and as a result, this book is an important contribution to the literature on explanatory models of HIV/AIDS in Africa.

